# Calibration of medical diagnostic classifier scores to the probability of disease

**DOI:** 10.1177/0962280216661371

**Published:** 2016-08-08

**Authors:** Weijie Chen, Berkman Sahiner, Frank Samuelson, Aria Pezeshk, Nicholas Petrick

**Affiliations:** Office of Science and Engineering Laboratories, Center for Devices and Radiological Health, Food and Drug Administration, Silver Spring, USA

**Keywords:** Calibration, classifier, rationality, probability of disease

## Abstract

Scores produced by statistical classifiers in many clinical decision support systems and other medical diagnostic devices are generally on an arbitrary scale, so the clinical meaning of these scores is unclear. Calibration of classifier scores to a meaningful scale such as the probability of disease is potentially useful when such scores are used by a physician. In this work, we investigated three methods (parametric, semi-parametric, and non-parametric) for calibrating classifier scores to the probability of disease scale and developed uncertainty estimation techniques for these methods. We showed that classifier scores on arbitrary scales can be calibrated to the probability of disease scale without affecting their discrimination performance. With a finite dataset to train the calibration function, it is important to accompany the probability estimate with its confidence interval. Our simulations indicate that, when a dataset used for finding the transformation for calibration is also used for estimating the performance of calibration, the resubstitution bias exists for a performance metric involving the truth states in evaluating the calibration performance. However, the bias is small for the parametric and semi-parametric methods when the sample size is moderate to large (>100 per class).

## 1 Introduction

Automated statistical learning classifiers are widely used in many medical applications. For examples, computer-aided diagnosis (CAD) algorithms are used in medical imaging for cancer detection,^1^ genomic classifiers are used to combine gene-expression data to predict patient response to therapy that can potentially lead to personalized treatments,^2^ clinical variables are combined using statistical models to predict the risk of breast cancer,^3^ among many others. In this work, we consider the binary outcome problem in which patients come from two populations (or truth states), e.g., diseased or non-diseased and responders or non-responders. Statistical learning algorithms often output numerical scores that are multi-level ordinal, quasi-continuous, or continuous, to which a cut-off threshold can be applied to predict the binary outcome. Many statistical classifiers yield scores on an arbitrary scale, the clinical meaning of which is often unclear. This may not be a problem if the scores are always dichotomized into binary decisions with a fixed threshold before being presented to physicians, i.e., the (quasi)continuous scores are not the final output. In many applications, however, ordinal, quasi-continuous, or continuous scores are output to physicians as they convey more information than dichotomized binary decisions. In such situations, it is important that the scores are on a meaningful scale so that physicians can understand and interpret them appropriately. In medical imaging, for example, CAD devices use statistical classifiers to output a score for a computer- or radiologist-detected region that represents the likelihood of the region being cancer. The radiologist can then incorporate this information in his/her diagnosis.

All classifier scores provide ranking information to physicians, e.g., patients with a score of 90 are more likely, on average, to be diseased than patients with a score of 55. It is not always clear, however, how likely a patient is diseased given a particular score without comparing with other patients. Moreover, it can be troublesome for a physician to interpret outputs from more than one diagnostic devices that output scores on arbitrary but different scales, since the same numerical score from different devices may have different meanings in terms of the likelihood that an abnormality of interest is present. It is therefore desired to transform classifier scores to a clinically meaningful scale. In this paper, we consider the probability of disease scale, although other scales such as the likelihood ratio scale are possible as well. It should be noted that there are methods to *normalize* classifier scores to the [0, 1] domain^4^; however, such normalized scores do not necessarily have a probability meaning and score normalization methods are *not* what we consider in this paper.

Our purpose in this research is to investigate methods for transforming classifier scores on arbitrary scales to the probability of disease scale. We begin with a review of related previous work. In the machine learning community, Platt^5^ proposed transforming support vector machines (SVM) scores to probability estimates by fitting a sigmoid function. Zadrozny and Elkan^6^ proposed an isotonic regression method implemented with the pool-adjacent-violators algorithm (PAVA).^7^ These two methods have been compared for a variety of classifiers.^8^ In the medical imaging community, Pesce et al.^9^ developed a method to transform classifier scores to the likelihood ratio scale based on a semi-parametric proper receiver operating characteristic (ROC) model.^10^ To the best of our knowledge, this method has not been compared with the other two methods. Moreover, the scale transformation function is estimated with a finite dataset and consequently the estimated probability has uncertainty that should be quantified. We initially presented a preliminary comparison of PAVA with the method extended from Pesce et al.^9^ at the SPIE Medical Imaging conference.^11^ In this paper, we extended our previous work to compare all three methods. Moreover, we developed techniques to quantify the uncertainty of the probability estimate for all three methods mentioned above. Finally, we evaluated and compared these methods using simulation studies and demonstrated their applications in a real-world CAD problem.

## 2 Methods

### 2.1 Relation between calibration and discrimination under the rationality assumption

It has been well known that probabilistic classifier scores have two fundamental properties: discrimination and calibration.^12^ Discrimination refers to the ability of a classifier to discriminate between two truth states, e.g., how likely the score of a diseased subject is greater than that of a non-diseased subject. In fact, a commonly used measure of discrimination, the area under the ROC curve (AUC), is exactly the probability that the score of a randomly chosen diseased subject is greater than that of a randomly chosen non-diseased subject.^13^ Calibration, on the other hand, describes the degree to which an estimated probability score agrees with the actual outcomes: out of 100 patients having the probability score of *x*%, it is expected that close to *x* of patients are actually diseased for a well-calibrated model. This paper is about transforming classifier scores to a calibrated probability scale. A natural question is whether this transformation would affect the discrimination.

Diamond^14^ pointed out that there is a trade-off between discrimination and calibration implying that a model maximizing one must be doing so by sacrificing the other. This notion has influenced other investigators^15^ and become somewhat popular. Unfortunately, this is a misconception. Diamond^14^ came up with this notion by “ proving” that any perfectly calibrated model can only achieve an AUC of 0.83. His proof, however, assumed that the probability scores are uniformly distributed in the whole population, which is neither realistic nor necessary for a statistical model to work. To disprove this trade-off misconception and further illustrate the relationship between discrimination and calibration, we provide a theoretical counter example below.

Let us assume that we have measurements of a set of *p* biomarkers (genomic, imaging, etc.) denoted by a *p* × 1 vector *X* and, conditioned on the truth state (*D* = 1 for diseased, and *D* = 0 for non-diseased), *X* follows multivariate normal distributions with a common covariance matrix *V* and means *μ*_1_ and *μ*_0_, respectively, i.e., *X*|*D* = 1 ∼ 𝒩(*μ*_1_, *V*), *X*|*D* = 0 ∼ 𝒩(*μ*_0_, *V*), where 𝒩(*μ*, *V*) denotes the density function of the normal distribution with mean *μ* and covariance matrix *V*. We further assume the prevalence of disease in the population is *η*. Consider a generalized linear classifier that consists of two steps: in the first step, *X* is mapped to a scalar variable *y* through a linear combination *y* = *w^T^X* where *w* is a *p* × 1 coefficient vector; in the second step, *y* is transformed to the probability of disease given *y* using a monotonically increasing function *P* = 1/(1 + exp(*Ay* + *B*)) with the constraint *A* ≤ 0. Since *y* follows a uni-variate normal distribution conditioned on the truth state and *P* is a monotonically increasing function of *y*, it can be shown that the AUC of this classifier is

(1) $$\text{AUC}=\Phi (\frac{w^{T}(\mu_{1}-\mu_{0})}{\sqrt{2w^{T}Vw}})$$

where Φ is the cumulative distribution function of the standard normal distribution. Moreover, we show (see Appendix 1) that *P* is perfectly calibrated probability of disease given *y* if and only if the parameters *A* and *B* are

(2) $$A=-\frac{w^{T}(\mu_{1}-\mu_{0})}{w^{T}Vw},\;B=\frac{w^{T}(\mu_{1}\mu_{1}^{T}-\mu_{0}\mu_{0}^{T})w}{2w^{T}Vw}-\log\frac{\eta }{1-\eta }$$

Note that the constraint *A* ≤ 0 is almost trivial because, if we have a classifier *w* such that *A* ≥ 0, we can always replace it with − *w*. Also note that *A* ≤ 0 implies *w^T^*(*μ*_1_ – *μ*_0_) ≥ 0 and hence 0.5 ≤ *AUC* ≤ 1.

In this theoretical example, we see that the discrimination ability of the classifier as measured by AUC in equation (1) is determined by the classifier coefficient parameters *w* and the intrinsic separability of the two populations given the measurements *X* that is characterized by parameters *μ*_1_, μ_0_, and *V*. Because the calibration function in step 2 is monotonically increasing, it does not have any effect on the discrimination. This means that, depending on the parameters, the AUC in equation (1) can be any value between 0.5 and 1 and, no matter what the AUC value is, the classifier scores can be perfectly calibrated to the probability of disease scale. The practical implication of this property is that the classifier coefficient vector *w* can be obtained by maximizing the AUC (i.e., discrimination) without considering calibration, which in this case is the Fisher's linear discriminant *w* = *V*^−1^(*μ*_1_ – *μ*_0_).^16^ And then, we can transform *y* to the probability of disease scale without affecting the discrimination in terms of AUC or any rank-based figures of merit.

In general, many classifiers (e.g., linear discriminant, SVM, and nearest neighbor classifiers) are trained by optimizing an objective function that is related to discrimination and produce classifier scores on some arbitrary scale that can then be calibrated to a meaningful scale. However, to calibrate scores without changing their ability to discriminate, the calibration function must be monotonically increasing. Thus, in this study, we make the rationality assumption where “rationality” is defined as a property that the classifier score and the probability of disease are related by a monotonically increasing function. We call this assumption the rationality assumption because it is *rational* to assign a higher score to a patient with a higher probability of disease. Rational classifier scores (*y*) must be a monotonically increasing function of the likelihood ratios of the scores (*LR*(*y*) = *f*(*y*|*D* = 1*)*/*f*(*y*|*D* = 0)), i.e., if *y*_1_ ≤ *y*_2_, then *LR*(*y*_1_) ≤ *LR*(*y*_2_). The proof is straightforward as follows. By the definition of rationality, *y* is a monotonically increasing function of the probability of disease *P*(*D* = 1|*y*). Moreover, the probability of disease *P*(*D* = 1| *y*) is monotonically related to the *LR* of *y* according to the Bayes' theorem: *P*(*D* = 1| *y*) = 1/(1 + *LR*^−1^ × (1 − *η*)/*η*). Therefore, *y* is a monotonically increasing function of *LR*(*y*). It is easy to see the converse is also true: if *y* is a monotonically increasing function of *LR*(*y*), then *y* is rational. Because of this property, the ROC curve for rational classifier scores is convex (called a proper ROC curve^17^).

We make a distinction between *rationality* and *optimality* of classifiers. Optimality of classifiers usually refers to optimal discrimination (e.g., AUC) given the measured attributes *X*. According to the signal detection theory,^17^ the optimal classifier (also known as the ideal observer) in terms of maximizing the AUC given *X* is the likelihood ratio of *X*. Clearly, the optimal classifier is rational. However, a rational classifier is not necessarily optimal. Referring to the theoretical example given above, the optimal classifier is the Fisher's linear discriminant and it is rational. But any linear classifier in this example that satisfies *w^T^*(*μ*_1_ −*μ*_0_) > 0 is rational. Rationality only requires the decision variable (*y*) be monotonically increasing with the likelihood ratio of itself (*LR*(*y*)), but not necessarily the likelihood ratio of the full attributes (*X*), which can be hard to obtain in practice without the knowledge of the joint distribution of the attributes. Because of this, the rationality assumption is not hard to meet in practice.

The rationality assumption allows one to calibrate classifier scores to the probability of disease scale without affecting the discrimination of the classifier and without even having to know how the classifier is trained. Under this assumption, we introduce three calibration methods.

### 2.2 Calibration method 1: The parametric method

Platt^5^ proposed a method for transforming scores (*y*) from SVMs to a calibrated posterior probability by fitting a sigmoid function

(3) P(D=1|y)=1/(1+exp(Ay+B))

where the parameters (*A* and *B*) can be obtained by solving a regularized maximum likelihood estimation (MLE) problem via a Levenberg-Marquardt algorithm.^18^ Lin et al.^19^ developed an improved algorithm in Platt's framework to overcome some numerical difficulties. In essence, this is just a uni-variate logistic regression method. The difference is that, instead of using the 0 and 1 target values as conventional logistic regression does, Platt^5^ applied a regularization to mitigate overfitting by using 1/(*N*_0_ + 1) and (*N*_1_ + 1)/(*N*_1_ + 2) as target values, where *N*_0_ is the number of actually negative subjects and *N*_1_ is the number of actually positive subjects. Since Platt's method assumes a parametric form of the scale mapping function, it is a parametric method. Although this method was proposed for calibration of SVM classifiers, it has been used for other classifiers as well^8^ since the method does not have to assume any specific classifier.

We implemented the algorithm by Lin et al.^19^ to estimate the parameters *A* and *B* given a set of scores with known truth states. Previous work in the literature did not include an estimation of the uncertainty of the estimated posterior probability. In this study, we adopted the delta method^20^ to compute the standard error of the probability estimate. Specifically, given a score *y*, the standard error of the estimated probability given *y*, *σ*_*P̂*_(*y*)__, where *P̂*(*y*) is the estimated probability, can be computed using the formula below with the MLE of *A* and *B* (denoted as *Â* and *B̂*, respectively)

(4) $$\sigma_{{\hat{P}}_{\left(y\right)}}=\sqrt{{\left(\frac{\partial P}{\partial A}\right)}^{2}\sigma_{\hat{A}}^{2}+{\left(\frac{\partial P}{\partial B}\right)}^{2}\sigma_{\hat{B}}^{2}+2(\frac{\partial P}{\partial A}\frac{\partial P}{\partial B})\mathrm{cov}(\hat{A},\hat{B})}$$

where the partial derivatives are derived analytically from equation (3), and the variances of *A* and *B* and their covariance are obtained from the inverse of the Hessian matrix output from the optimization procedure.

### 2.3 Calibration method 2: The semi-parametric method

Pesce et al.^9^ developed a method for transforming classifier scores to the log-likelihood ratio (LLR) scale. Their method is closely related to the proper ROC model developed by Metz and Pan.^10^ In their method for fitting a proper ROC curve, the classifier scores are assumed to be monotonically related to a latent continuous decision variable that is rational. Specifically, they used an explicit monotonic transformation of the likelihood ratio of normal distributions as a latent decision variable (denoted as *u*). In their algorithm, classifier scores are first sorted and categorized into I bins using truth state runs as described in Metz et al.^21^ (rather than ad-hoc binning), where *I* depends on the data. These bins are mapped to the latent space with subject-occurrence frequency in each bin the same as that in the corresponding bin in the score space thanks to the monotonicity assumption. The likelihood for observing these binning data (i.e., numbers of subjects in the bins) is a multinomial function of the following latent-space parameters: two parameters for the normal distributions, *I* – 1 boundary parameters for *I* bins. An MLE procedure is used to estimate these parameters from which the ROC curve and ROC-based accuracy indices are derived and their standard errors are computed from the Hessian matrix output by the MLE procedure. The readers are referred to the Metz and Pan^10^ for more technical details. Note that the approach is deemed to be semi-parametric instead of parametric because the classifier scores are not assumed to follow a specific parametric form but are assumed to be related to a latent decision variable through an implicit monotonic transformation.

Using the estimated distribution parameters and boundary parameters for the bins, a method in Metz et al.^21^ allows for the estimation of boundary values on the latent axis for each distinct value in the test score. Note that each distinct value in the test score may correspond to a single subject or, when there are ties in the data, a number of subjects with one or mixed truth states. Suppose that, for the *i*th distinct test value *y_i_*, the boundary values on the latent axis are *u*_*i*−1_ and *u_i_* and there are *n*_*i*0_ actually negative subjects and *n*_*i*1_ actually positive subjects tied for *y_i_*. Then using these parameters together with the distribution parameters, Pesce et al.^9^ chose to use the median value between *u*_*i*−1_ and *u_i_* as the *complementary* value *ũ* in the latent space for the test value *y_i_*. Because there is an explicit monotonic relationship between *u* and the LLR defined in the proper ROC model, the LLR corresponding to *ũ* can thus be computed. The associated standard error can then be computed via the delta method.^9^

Assuming a known prevalence of disease *η*, the LLR obtained via the semi-parametric method of Pesce et al.^9^ can then be transformed to the posterior probability using the Bayes theorem

(5) $$P(D=1|y)=\frac{\eta e^{\text{LLR}(y)}}{\eta e^{\text{LLR}(y)}+(1-\eta )}$$

By replacing LLR with its estimate $\hat{\text{LLR}}$, we obtain an estimate of the posterior probability. The standard error of the estimated probability is computed with the delta method similar to that in the previous method.

### 2.4 Calibration method 3: The non-parametric method

A non-parametric isotonic regression method can be used to calibrate classifier scores to the probability of disease as demonstrated by Zadrozny and Elkan.^6^ Given a dataset ${\left(y_{i},D_{i}\right)}_{i=1}^{N}$ of *N* subjects with the *i*th subject having a classifier score *y_i_* and the truth state *D_i_* ∈ {0, 1}, isotonic regression finds, from the family of isotonic functions (*z*), the function *g* that maps the classifier score *y_i_* to the posterior probability that *y_i_* belongs to the diseased class, *g*(*y_i_*) ≡; *P*(*D_i_* = 1|*y_i_*), i.e.

(6) $$g={\mathrm{argmin}}_{z}\sum {\left(D_{i}-z(y_{i})\right)}^{2},\;\text{subject to}g(y_{i})\leq g(y_{i\prime })\text{if}y_{i}\leq y_{i\prime }$$

A widely used algorithm for isotonic regression computation is the PAVA.^7^ This algorithm works as follows. First, the classifier scores are sorted ascendingly: ${\left(y_{i}^{*},D_{i}^{*}\right)}_{i=1}^{N},\;y_{i}^{*}\leq y_{i^{\prime }}^{*}$ if *i* ≤ *i′*. The probability estimate for each subject is initialized with its binary truth state: $g^{\left(0\right)}(y_{i}^{*})=D_{i}^{*}$ and each subject is put in its own group *G_i,i_*. The notation *g*^(*j*)^ denotes the probability estimate at the *j*th iteration and *G_i,i′_* denotes the group of (sorted) subjects from subject *i* to subject *i′*(*i* ≤ *i′*). Then the following is repeated iteratively: in the *j*th iteration, search from the beginning until the first occurrence of a pair of groups *G_k,i_*_–1_ and *G_i,l_* such that $g^{j-1}(y_{i-1}^{*})>g^{j-1}(y_{i}^{*})$ (adjacent violator), then pool the subjects in *G_k,i_*_–1_ and *G_i,l_* into one group *G_k,l_* and assign $g^{\left(j\right)}(y_{i}^{*})=(\sum_{i=k}^{l}g^{\left(j-1\right)}(y_{i}^{*})/(l-k+1))$ for *i* = *k*, …, *l* and $g^{\left(j\right)}(y_{i}^{*})=g^{\left(j-1\right)}(y_{i}^{*})$ for *i* < *k* and *i* > *l*; if no adjacent violators, then stop. An illustrative example of the PAVA is shown in Table 1.

We used the bootstrap approach^22^ to estimate the confidence intervals (CIs) of the PAVA estimated probability. Specifically, we bootstrap the classifier score dataset ${\left(y_{i},D_{i}\right)}_{i=1}^{N}$ many times (in this paper, we set *B* = 2000 times) using a balanced bootstrap, i.e., each subject is sampled exactly the same number of times. For each bootstrapped dataset ${\left(y_{i}^{b},D_{i}^{b}\right)}_{i=1}^{N}$, we apply the PAVA to obtain an estimate of the probability of disease for each subject in the bootstrap dataset. Then, by aggregating all the estimates, each classifier score in the original dataset has *B* estimates of the probability of disease, from which a bootstrap percentile CI is constructed.

### 2.5 Remarks on calibration methods: Assumptions, training, and testing

All the three calibration methods described above share the rationality assumption regarding the score data. This is clear for Platt's parametric method as it assumes an explicit monotonic relation (i.e., the logistic function) between the score and the probability. Moreover, the specific logistic function as defined in equation (3) is the correct model when the scores follow certain distributions, e.g., a pair of normal distributions with equal variance (as shown in Section 2.1) or a pair of exponential distributions.^5^ However, it is not necessarily the correct model for other types of distributions. For example, it can be verified that the logistic model is not the correct model for the beta distribution score that we use for simulation in the next section. In the semi-parametric method, the score data are not required to follow specific distributions but is assumed to be monotonically related to the likelihood ratio of normal distributions. The non-parametric method makes the rationality assumption by the constraints in the optimization but makes no further assumption regarding the distribution of the score data. It is interesting to note that, similar to the connection between the semi-parametric calibration method and the semi-parametric proper ROC model, the PAVA-generated probability scores correspond to the so-called ROC convex hull,^23,24^ which is essentially a non-parametric estimate of the proper ROC curve.

**Figure 1:**
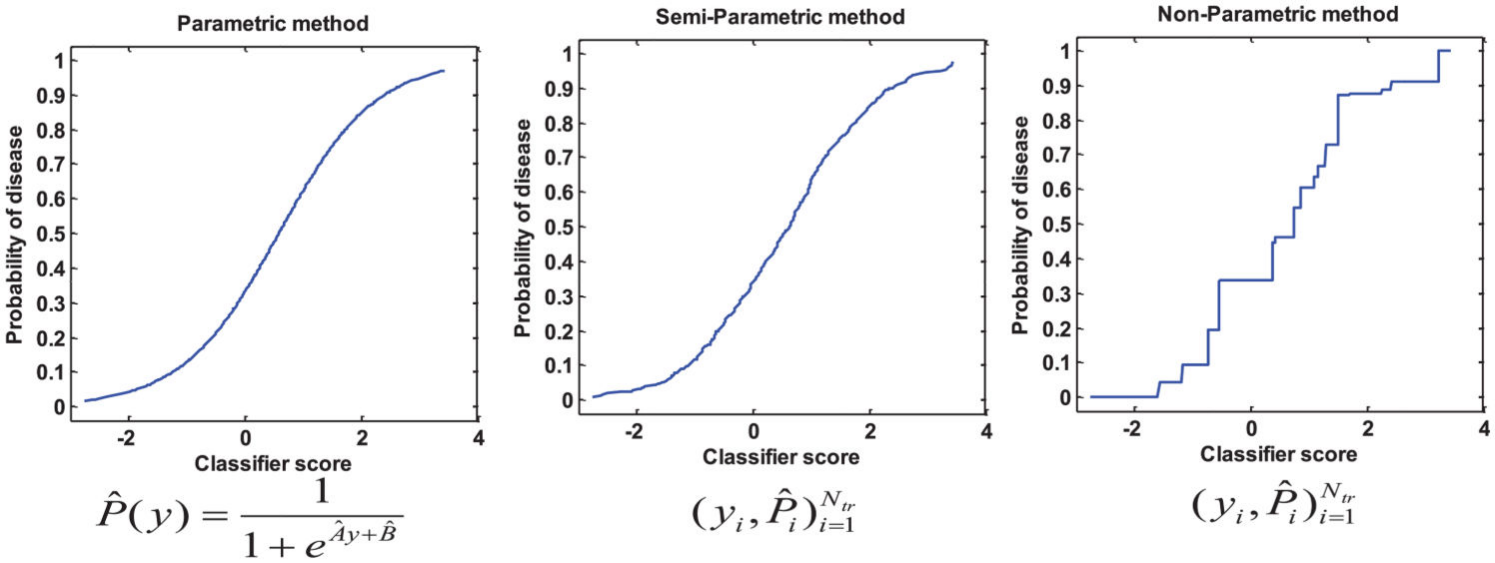
Examples illustrating training of calibration functions: the parametric method gives a functional formula with parameters estimated from the training data; the semi- and non-parametric methods give an estimate of the probability of disease (*P̂_i_*) for each score (*y_i_*) in the training data.

These calibration methods require a training dataset *o*f *N_tr_* score samples with truth states to determine the score-to-probability transformation function (which we call the calibration function from now on). As illustrated in Figure 1, the calibration function in the parametric method is a sigmoid function characterized by two parameters *A* and *B* and training gives an estimate of these two parameters *Â* and *B̂*. In testing on a new score *y*, the probability can be calculated using the trained formula (shown in Figure 1 below the first graph). For the semi-and non-parametric methods, the training yields an estimate of the probability of disease for each score in the training data. The transformation function is thus defined on these discrete training data points. In testing on a new score *y*, we use interpolation on the training data to obtain the probability estimate.

### 2.6 Evaluation

The evaluation of algorithm-based medical diagnosis devices has predominately relied on rank-based metrics such as sensitivity, specificity, the AUC, among many others.^25,26^ Such measures characterize the discrimination or classification ability of a device. However, they do not capture the calibration or scale information of classifier scores because, for example, a change of the scale of classifier scores by a monotonic transformation would not change the aforementioned rank-based metrics. Performance metrics that are dedicated to or account for calibration have been an active area of research in recent years; however, there is a lack of consensus on which metrics are the best and controversies exist for at least some recently proposed metrics.^27–30^ A comparison of different performance metrics is interesting, but is beyond the scope of this paper. Instead, we consider a classic performance metric, namely the Brier score (BS)^31^, to investigate the properties of the three calibration methods as presented above. The Brier score is defined as the mean square difference between the probability (*P*) and the truth state (*D*: 0 or 1) and thus accounts for both discrimination and calibration. Given a dataset of *N* subjects, the BS can be estimated as

(7) $$\hat{\text{BS}}=\frac{1}{N}\sum_{i=1}^{N}{\left({\hat{P}}_{i}-D_{i}\right)}^{2}$$

We used simulations to investigate the statistical properties of the three calibration methods. One advantage of using simulation is that it allows a direct comparison of the estimated probabilities with the true probability of disease that can be computed analytically. Specifically, two simulation models were used to generate classifier score data. The first model was a pair of normal distributions with different means and equal variance. We chose parameters corresponding to an AUC value of approximately 0.80, i.e., *y*|*D* = 0 ∼𝒩(0, 1), *y*|*D* = 1 ∼ 𝒩(1.2, 1). As mentioned earlier, the decision variable *y* with such density functions is rational. The second model was a pair of beta distributions

(8) $$\begin{array}{cccc} f(y|D=0)=\beta {\left(1-y\right)}^{\beta -1}, & \beta \geq 1; & ,f(y|D=1)=\alpha y^{\alpha -1}, & \alpha \geq 1 \end{array}$$

Note that the density function of the beta distribution generally has two parameters, and the density functions in equation (8) are special cases by setting one of the parameters to be 1 and setting the other to be greater than or equal to 1. As shown by Mossman and Peng,^32^ the decision variable *y* with this particular form of beta density functions has a “proper” ROC curve, i.e., *y* is rational. In this study, we set *α* = 1.1, *β* = 3.5 that yields an AUC value of approximately 0.80.

Since the probability density functions of *y* are known, the true posterior probability of disease (*D*= 1) can be calculated as

(9) $$P(D=1)=\frac{\eta f(y|D=1)}{\eta f(y|D=1)+(1-\eta )f(y|D=0)}$$

where the disease prevalence *η* is assumed to be known. A summary statistic, namely the mean square error (MSE), can be computed from a sample of *N* subjects

$$\hat{\text{MSE}}=\frac{1}{N}\sum_{i=1}^{N}{\left({\hat{P}}_{i}-P_{i}\right)}^{2}$$

In our simulations, we randomly sampled a training dataset from the specified distributions and trained the calibration functions in the three methods. This yielded, for each method, a calibration function and the associated 95% CI. The estimated calibration function was compared with the true calibration function that was calculated using equation (9). The average length of the 95% CI was computed as a surrogate measure of the variability of the calibration function.

We computed the two performance metrics, namely the Brier score and the MSE, under two evaluation scenarios. In the first scenario, we used the probability estimates of the training data to compute BS and MSE. This is equivalent to training and testing the calibration function using the same dataset and we call it the *resubstitution evaluation*. In the second scenario, we used the probability estimates of an independently drawn dataset to compute BS and MSE, which we call the *independent evaluation*.

## 3 Simulation results

**Figure 2:**
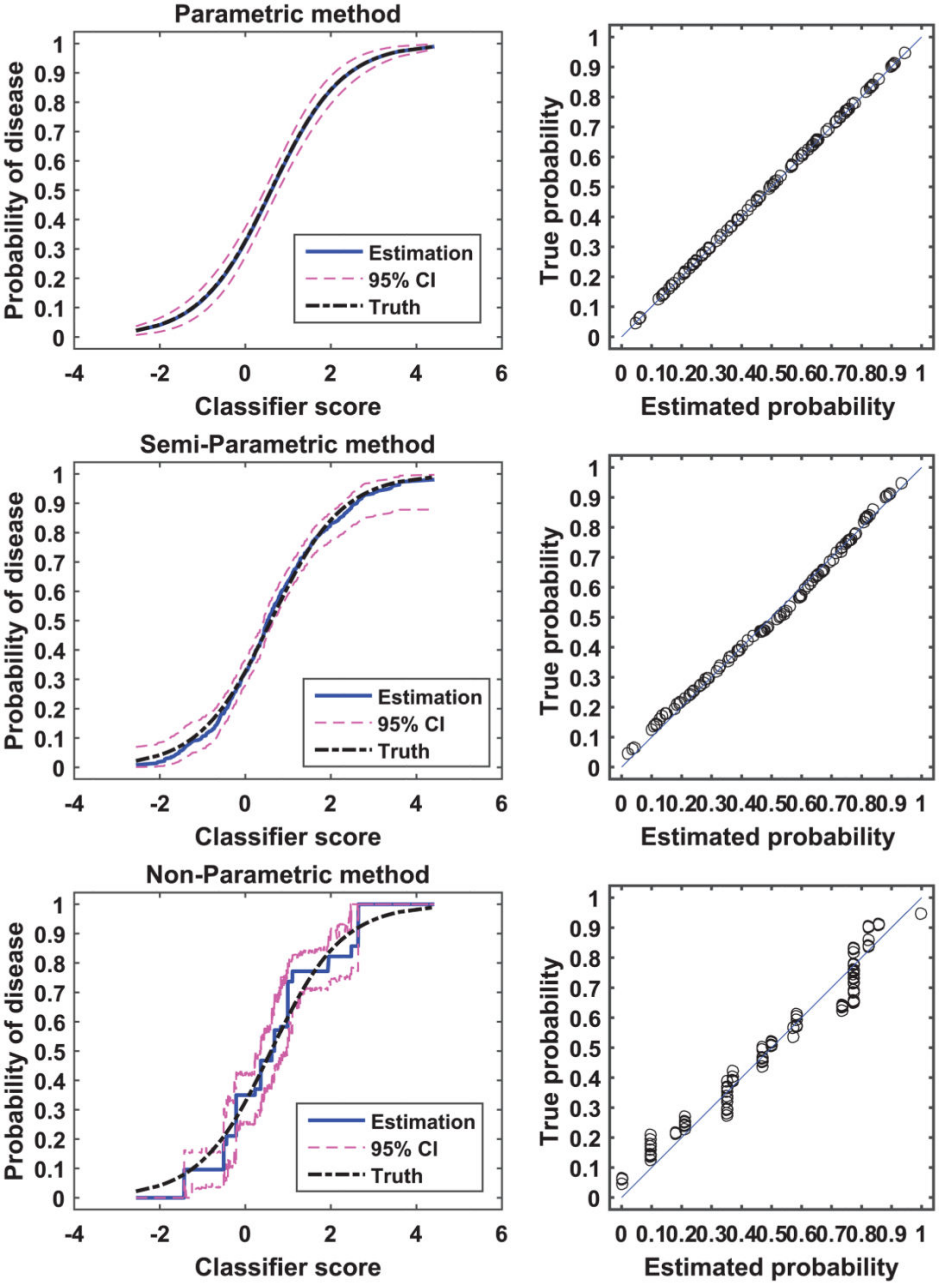
An example of calibration. The two-class score data were generated from a pair of normal distributions with 300 samples per class. The left panel plots the true calibration function (dot-dash line), the estimated calibration function (solid line) and the associated 95% CI (dash line). The right panel plots the true versus estimated probability for the finite dataset.

**Figure 3:**
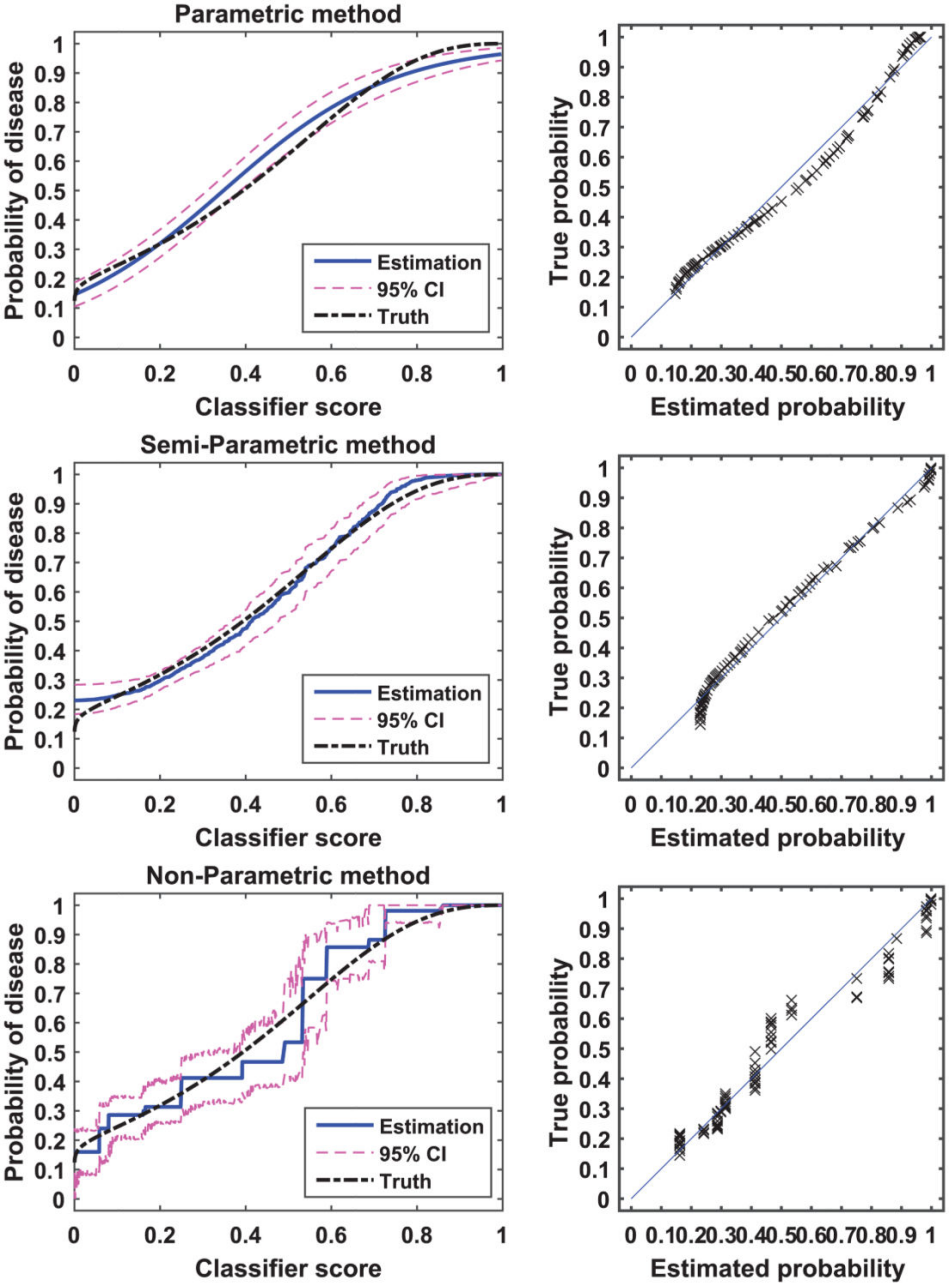
An example of calibration. The two-class score data were generated from a pair of beta distribution with 300 samples per class. The left panel plots the true calibration function (dot-dash line), the estimated calibration function (solid line) and the associated 95% CI (dash line). The right panel plots the true versus estimated probability for the finite dataset.

Figures 2 and 3 show examples of calibration by the parametric, semi-parametric, and non-parametric methods for the normal distribution and the beta distribution, respectively. In each of the two examples, we sampled 300 subjects per class from the respective distributions. In these two figures, we plot the calibration function, i.e., the probability of disease versus the classifier score, for the three methods and for the analytical truth. In addition, we provide scatter plots of the true versus estimated probability for each method. By comparing the two figures, we see that the parametric method appears to be more sensitive to the distribution of the score data than the other two methods: the parametric method calibration appears to be perfect for the normally distributed data, but discrepancies between the estimated and the true calibration function are evident for the beta distribution data (bias). Moreover, we observe that the parametric and the semi-parametric methods appear to have narrower CI than the non-parametric method.

**Figure 4:**
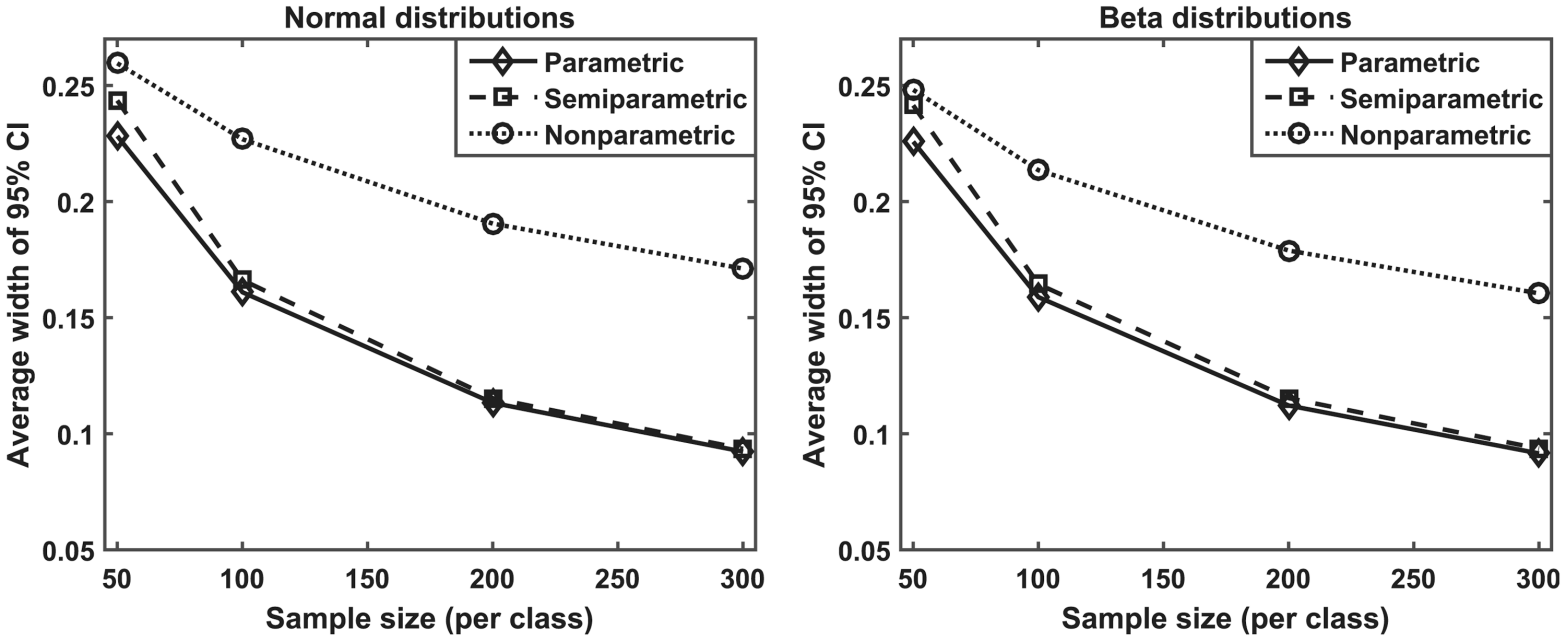
The average width of the 95% CI as a function of sample size for the three methods and for the normal distribution data (left) and beta distribution data (right) respectively.

More quantitative comparisons are made using repeated simulation experiments. In this work, we varied the sample size for estimating the calibration functions (from 30 or 50 subjects per class, to 100, 200, and 300 subjects per class). For each sample size, we repeated the simulation 800 times by generating score data independently in each repetition. Figure 4 plots the average width of the 95% CI as a function of sample size for the three methods and for the normal distribution data (left) and beta distribution data (right), respectively. The average width of the 95% CI is a surrogate measure of the variability of the estimated calibration function. Figure 4 indicates that, as expected, the variability decreases as the sample size increases. More importantly, the results indicate that the semi-parametric and the parametric method have similar levels of variability and they are substantially lower than that of the non-parametric method.

**Figure 5:**
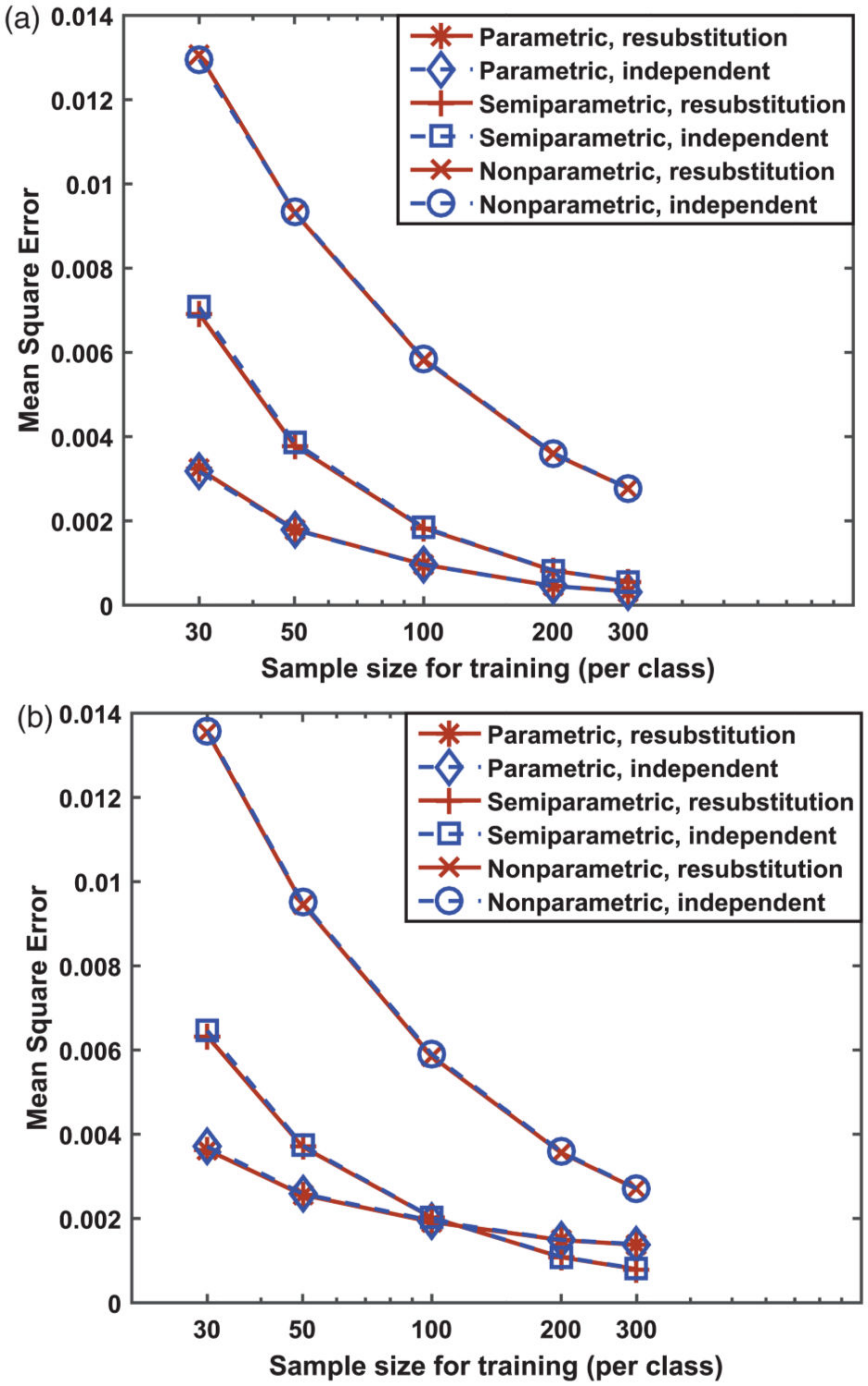
Mean square error of calibrated probabilities with respect to the true probabilities for (a) normal distribution data, and (b) beta distribution data.

The lower variability of the parametric and semi-parametric methods may be expected to result in paying a price in bias as the bias-variance trade-off usually plays out. To this end, we examined the MSE of the probability estimates. The results, as shown in Figure 5, indicate that, for all the sample sizes examined and for both types of distributions, the non-parametric method has substantially larger MSE than the parametric and semi-parametric methods. The relative ranking of the parametric and the semi-parametric methods was found to depend on the distribution of the data. For normally distributed score data, the parametric method has the best (i.e., lowest) MSE under all the sample sizes examined, as shown in Figure 5(a). This is expected because, as mentioned earlier, the parametric model is the correct model for equal-variance normal distributions and parametric estimation is generally known to be most efficient. For score data, following the beta distribution, however, the MSE versus sample size curves cross for the parametric and semi-parametric methods, as shown in Figure 5(b). Given that the variance is similar between the parametric method and the semi-parametric method and it is similar between the two types of data distributions (Figure 4), we infer from the MSE results that the parametric method is more sensitive to the type of data distribution in terms of bias than the semi-parametric method (i.e., the bias for the semi-parametric method is similar across data distributions whereas the bias for the parametric method is larger for beta distribution data than for normal distribution data). The “cross” in Figure 5(b) is the usual bias-variance trade-off phenomenon: at large sample sizes (200 and 300 per class), the variance is similarly low for both methods (Figure 4, left) and the larger MSE of the parametric method (Figure 5(b)) is attributed to its larger bias; at smaller sample sizes (30 and 50 per class), the variance is high for both methods and dominates the MSE and the relatively larger variance of the semi-parametric method contributes to its larger MSE.

**Figure 6:**
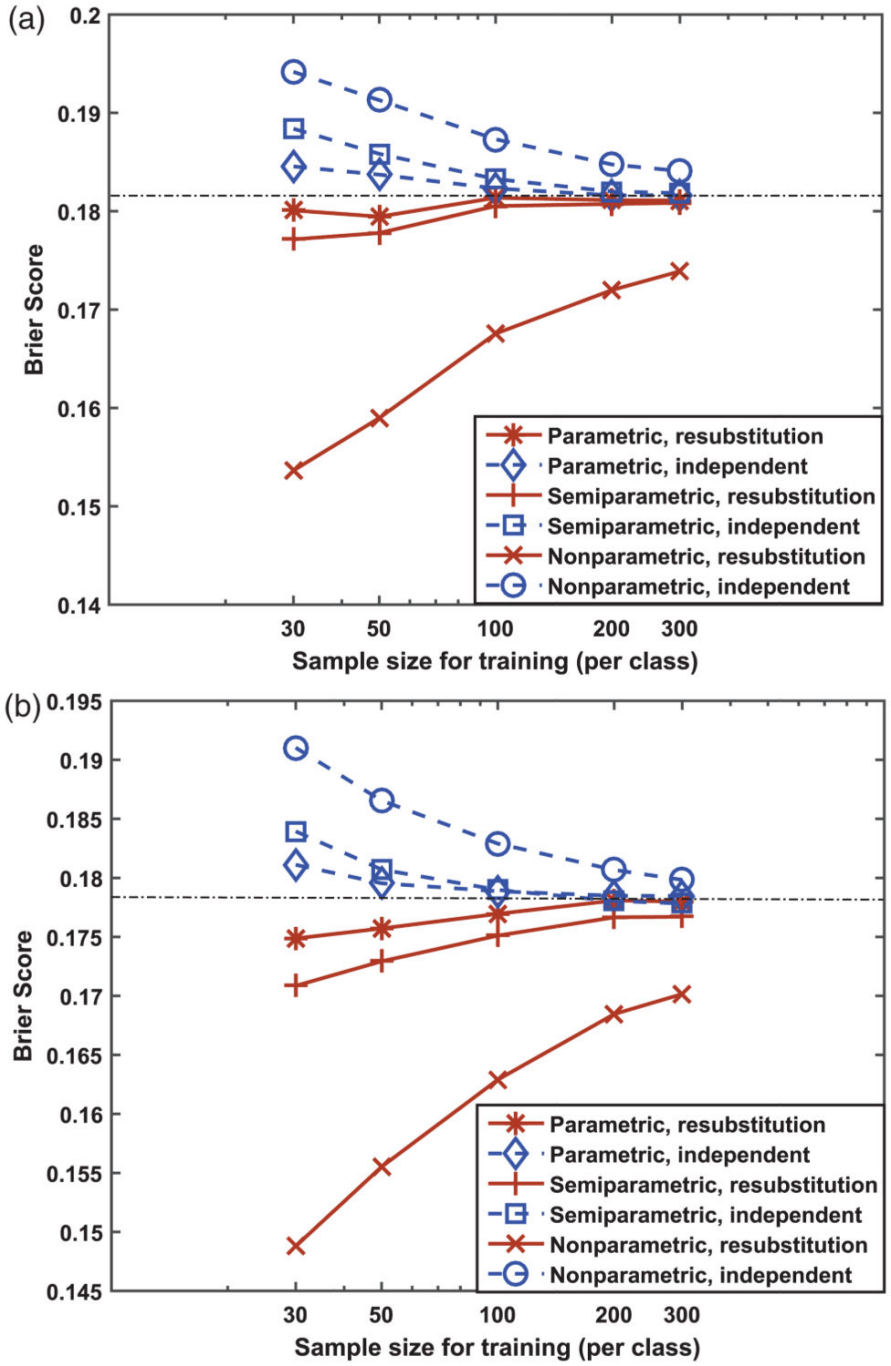
Brier score of calibrated probabilities for (a) normal distribution data, and (b) beta distribution data. The horizontal dot-dash line corresponds to the Brier score for perfectly calibrated scores (or infinitely trained calibration function).

Finally, the results of our simulations investigating the effect of the resubstitution versus independent testing on summary performance metrics (MSE and BS) are presented in Figures 5 and 6. Figure 6 shows, in reference to the BS of the infinitely trained (or theoretical) calibration function (plotted as dot-dash lines in Figure 6, BS = 0.1814 for normal data and *BS* = 0.1783 for beta data obtained by numerical computation using large samples), the resubstitution estimates of BS are optimistically biased and the independent estimates of BS are pessimistically biased. This phenomenon is similar to the training of classifiers.^33^ In addition, the two estimates appear to be converging as the training sample size increases and the parametric and semi-parametric methods seem to converge faster than the non-parametric method. We emphasize that the bias mentioned above is in reference to the infinitely trained calibration function, whereas the independent estimate of BS is unbiased by definition for a finite-trained and fixed calibration function's population performance.

By contrast, the resubstitution versus independent testing seems to have no effect on the MSE, as shown in Figures 5. This can be explained by the fact that the truth state values, but not the true probability values, are involved in training of the calibration function for all the three methods. For example, the objective function in the non-parametric method is in fact the Brier score (see equations (6) and (7)). The training procedure minimizes the objective function, which effectively minimizes the BS *for the training data* by driving the probability of non-diseased subjects towards 0 and the probability of diseased subjects towards 1, but it is not necessarily generalizable to the independent testing data. However, this does not happen to the MSE because the true probability values are not involved in training and there is no self-consistency between the MSE and the training data.

## 4 A real-world example

**Figure 7:**
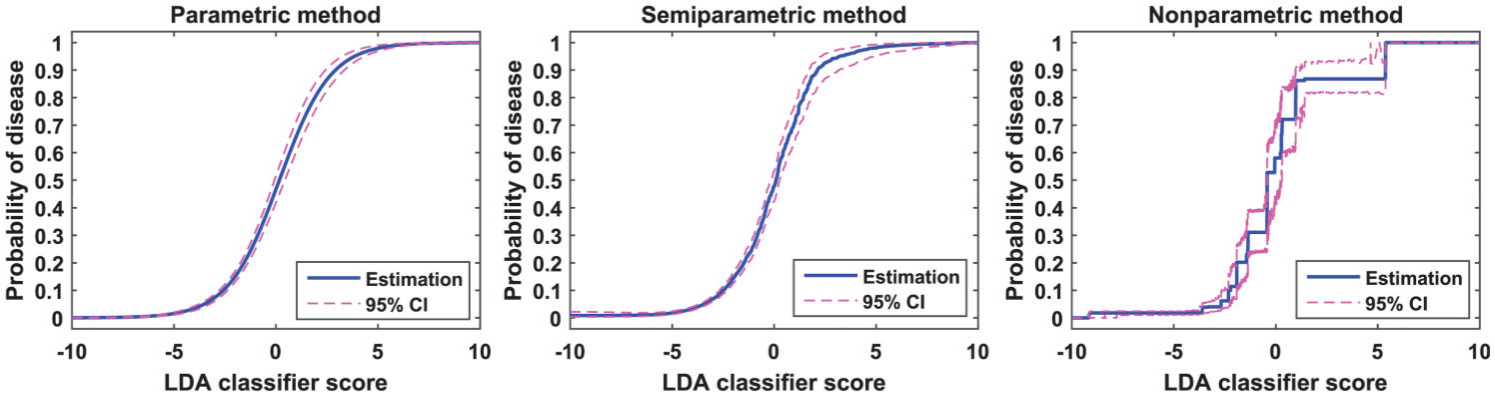
Calibration of LDA classifier scores to the probability of being a nodule.

We demonstrate the three calibration methods on a medical image CAD application. In this application, a computer algorithm was developed to detect lung nodules in computed tomography images. Image analysis algorithms were used to detect candidate regions of interest (ROIs) from the images. Then characteristic image features were extracted to distinguish between nodules and non-nodules. A linear discriminant analysis (LDA) classifier was trained using these image features to classify the ROIs into nodules or false detections. In the test dataset, we have 360 nodules and 2314 non-nodules. The LDA scores for these test cases are uncalibrated and thus are not clinically meaningful. We transform them to the probability of disease (i.e., nodule) using the three methods investigated in this paper. Figure 7 shows the calibration curves with the associated 95% CI. The average width of the 95% CI for the parametric, semi-parametric, and non-parametric method is 2.6%, 2.8%, and 5.5%, respectively. The resubstitution and leave one out cross validation (CV) estimates of the Brier score are presented in Table 2. These results show that, with the reasonably large dataset in this application, the calibration function is estimated accurately by all three methods. The resubstitution and CV estimates of the BS metric are nearly the same.

## 5 Discussions

In this paper, we investigated three methods for calibrating classifier scores on an arbitrary scale to the probability of disease scale. We examined the assumptions behind these methods and developed uncertainty estimation techniques. We then investigated their statistical properties using simulation studies: variability, MSE with respect to the true probability of disease, Brier score, and their interplays with sample size and the type of distribution of the data. We showed that, under the rationality assumption, classifier scores on arbitrary scales can be calibrated to the probability scale without affecting discrimination.

Intuitively, one may expect that a huge number of samples are needed to reliably calibrate classifier scores to the probability scale. However, our simulation results show that, with the principled methods investigated in this paper, a sample size of a few hundreds can achieve a calibration with reasonably favorable properties (e.g., 95% CI length of around 10 – 15 % as shown in Figure 4). This is essentially because, by using models, we do not have to calibrate every probability value independently. Of course, the larger the sample size, the more accurate the calibration. It is therefore important to accompany the probability estimate with an appropriately calculated CI. We argue that, even if the dataset for calibration is limited and hence the CI might be wide, a probability estimate with the CI may be more informative than a score on a meaningless arbitrary scale.

Our simulations show that there might be no universal winner among the three calibration methods investigated. The parametric method enjoys lower variability; however, it may not be sufficiently robust to different types of distributions of the score data. The non-parametric method seems to be robust to different data distributions but it suffers from large variability. Based on our simulations, the semi-parametric method may be a reasonable choice for many applications as it seems to have an acceptable trade-off between robustness and precision—it is not as sensitive to the data distribution as the parametric method and it is substantially less variable than the non-parametric method. One would need to examine the nature of the data (type of distribution, sample size, etc.) to choose an appropriate method for a particular application. With a sufficiently large sample size, we have found that all the three methods work pretty well.

In medical applications, patient sample size is often limited as compared to sample sizes in other industrial pattern recognition applications. This is due to a variety of factors such as low disease prevalence, the cost of collecting medical data, and the inability or unwillingness of some patients or research groups to share data, etc. This has to be taken into account in designing and validating classifiers for medical diagnosis. Ideally, one would wish to have independent datasets for training, calibration, and testing of performance. However, this may not be the only or even the best way with limited resources available because partitioning a limited dataset into independent subsets may render each of them too small to do meaningful training and testing. It is well established that it is crucial to use independent datasets for training and testing a multi-variate classifier because of the severe bias in resubstitution. However, as our simulation results show, the resubstitution versus independent testing bias for a calibration function is not as severe with a reasonable sample size, which at least in part is because classifiers are multi-dimensional and the calibration function is uni-variate and resubstitution bias (or lack of generalizability) becomes worse at higher dimensions (a phenomenon known as the curse of dimensionality).^34,35^, This, together with the fact that calibration is independent of discrimination, implies that one may be able to use one dataset for training the classifier to optimize the discrimination ability of the classifier, and then use an independent test set to both test the discrimination performance and train the calibration function. To ensure a rigorous assessment, one can use CV of the latter dataset to assess the calibration performance.

It should be emphasized that the diseased samples and non-diseased samples in the dataset for training the calibration function should be representative of the diseased and non-diseased populations, respectively. However, in general, the sample prevalence of disease (*η_sample_*) is not required to be the same as the population prevalence (*η_pop_*). This is because, when *η_sample_* ≠ *η_pop_*, one can scale the probability of disease estimated from the samples to that in the population.^36^ Note that we have *P*(*D* = 1| *y*) = *ηLR*(*y*)/(*ηLR*(*y*) + 1 – *η*) where the likelihood ratio *LR*(*y*) is independent of the prevalence. Moreover, *LR*(*y*) in the sample dataset is asymptotically the same as the *LR*(*y*) in the population (subject to random sample variations) as long as the diseased or non-diseased samples are random samples (hence representative) of their respective populations. Therefore, the probability of disease estimated from the samples *P̂_sample_* can be scaled to the estimated probability in the population *P̂_pop_* to match the population prevalence by

(10) $${\hat{P}}_{\text{pop}}=\frac{\kappa {\hat{P}}_{\text{sample}}}{(\kappa -1){\hat{P}}_{\text{sample}}+1}$$

where

$$\kappa =\frac{\eta_{\text{pop}}/(1-\eta_{\text{pop}})}{\eta_{\text{sample}}/(1-\eta_{\text{sample}})}$$

So far, we have assumed that classifier scores are on an arbitrary scale. We should point out that some algorithms (e.g., logistic regression, Gaussian process classifiers^37^ and Bayesian neural networks^38^) output scores directly on the probability scale and a post-calibration procedure may be unnecessary. However, the probability scores output by such algorithms correspond to the prevalence of algorithm training samples and they can be converted to probability scores for a target population prevalence value using equation (10).

Although we have focused on automated classifiers in our discussions of the calibration methods, the methods developed and evaluated in this study have broader applications. The classifier scores discussed in this paper can be from automated algorithms, but they can be from human classifiers or physical measurements of a biomarker as well. For example, Horsch et al.^39^ used the likelihood ratio scale^9^ as an intermediate vehicle to transform scales between scores of computer algorithms and ratings of radiologists in image-based cancer detection tasks, which has the potential to help radiologists better understand and use the computer output. For another example, Pepe^26^ cautioned against pooling the rating scores of multiple radiologists for data analysis as they may use different scales. Use of calibration methods to unify their scales may help mitigate this concern.

Finally, we note that there are two potentially important extensions of the current work. The first is the evaluation of calibration. In this work, we used the Brier score to evaluate the training and testing of calibration functions (e.g., resubstitution bias). However, the Brier score may not be sufficiently informative for real-world applications because it combines both the discrimination and the calibration performance into one number. In practical applications, it is often desired to evaluate the two types of performance information separately. Discrimination measures such as AUC, sensitivity, and specificity are well established. More research is needed to investigate properties of different measures (e.g., calibration slope and Hosmer–Lemeshow test as investigated in Steyerberg et al.^27^) and develop consensus for the most appropriate measure of calibration in practical clinical applications.

The second potential extension of the current work is to investigate methods for setting thresholds that warrant appropriate clinical actions. Note that even though a model is trained based on two-truth-state data, the patients can be categorized into more than two groups based on continuous classifier scores for appropriate treatments (e.g., sending home for healthy patients, follow-up for intermediate patients, and therapeutic treatment for diseased patients). One problem of setting thresholds based on observed classifier scores is that the thresholds may be set to optimize certain performance measure on the same dataset that is used to evaluate the performance (e.g., sensitivity) thereby introducing bias to that performance estimate.^40^ A potential solution is to set thresholds on the (absolute) probability scale, which can be done in the data analysis plan before collecting the data thus avoiding bias of the data-dependent method. Such thresholds can then be transformed through a score-to-probability calibration function to the classifier score scale. Another advantage of setting thresholds on the probability scale is that it is straightforward to apply utility (or cost) analysis on that scale. With utilities defined for each clinical action and truth state combination, the thresholds are determined by maximizing the expected total utility. For example, in a two-truth-state problem, there are four utilities, defined respectively for true positives, false negatives, true negatives, and false positives as *U_TP_*, *U_FN_*, *U_TN_*, and *U*_FP_, and the optimal threshold on the posterior probability can be found as a function of the utilities. Incidentally, a “50% chance” threshold is not necessarily optimal unless the relative utility, defined as (*U_TP_* – *U_FN_*)/(*U_TN_* – *U*_FP_), is unity.

## 6 Conclusion

In conclusion, we investigated three methods for calibration of classifier scores to the probability of disease scale and developed uncertainty estimation techniques for these methods. Under the rationality assumption, classifier scores on arbitrary scales can be calibrated to the probability of disease scale without affecting discrimination. With a finite dataset to train the calibration function, it is important to accompany the probability estimate with its CI. Our simulations indicate that the resubstitution bias exists for a performance metric involving the truth states in evaluating the calibration performance, but the bias is small for the parametric and semi-parametric methods when the sample size is moderate to large (>100 per class).

## Figures and Tables

**Table 1:** An illustrative example of the pool adjacent violators algorithm.

Subject	y*	*g*^(0)^: step 0 (D*)	*g*^(1)^: step 1	*g*^(2)^: step 2	*g*^(3)^: step 3	*g*^(4)^: step 4
8	2	0	0	0	0	0
4	12	1	1/2	1/3	1/3	1/3
1	18	0	1/2	1/3	1/3	1/3
6	20	0	0	1/3	1/3	1/3
7	27	1	1	1	1	2/3
9	30	1	1	1	1/2	2/3
2	42	0	0	0	1/2	2/3
3	50	1	1	1	1	1
10	55	1	1	1	1	1
5	78	1	1	1	1	1

The scores (*y*) are sorted ascendingly (y*) and the probabilities are initialized with the truth states D* (step 0). In each step, the adjacent violators (i.e., the order of the probabilities is opposite to that of the scores) are pooled into one group (boxes in the table) and the estimated probabilities in the box are averaged before going to the next step until no violators are found.

**Table 2:** Brier score results for the calibrated LDA classifier in lung nodule detection.

	Parametric	Semi-parametric	Non-parametric
Resubstitution	0.0590	0.0583	0.0557
Leave one out cross validation	0.0592	0.0586	0.0578

## Appendix 1

### Calibration function of linear classifiers for multi-variate normal data

Following the notation in Section 2.1, assuming we have *p*–dimensional normal data: *X*|*D* = 1 ∼ 𝒩(*μ*_1_, *V*), *X*|*D* = 0 ∼ 𝒩(*μ*_0_, *V*), where 𝒩 (*μ*, *V*) denotes the density function of the normal distribution with mean *μ* (a vector of *p* × 1) and covariance matrix *V* of size *p* × *p*. Using a linear combination, *X* is mapped to a scalar variable *y*: *y* = *w^T^X*. Our goal is to find *P*(*D* = 1|*y*), the probability of disease given score *y*. Note that the conditional distribution of *y* is uni-variate normal, i.e.

(11) $$y|D=1\sim N(w^{T}\mu_{1},w^{T}V_{w}),\;y|D=0\sim N(w^{T}\mu_{0},w^{T}V_{w})$$

According to the Bayes theorem, we have

$$P(D=1|y)=\frac{P(D=1)f(y|D=1)}{P(D=1)f(y|D=1)+P(D=0)f(y|D=0)}$$

By inserting the conditional density functions in equation (11) into the equation above and also note that the prevalence of disease *η* ≡ *P*(*D* = 1) is assumed to be known, we have

$$P(D=1|y)=\frac{1}{1+\exp(Ay+B)}$$

where

$$A=-\frac{w^{T}(\mu_{1}-\mu_{0})}{w^{T}Vw},\;B=\frac{w^{T}(\mu_{1}\mu_{1}^{T}-\mu_{0}\mu_{0}^{T})w}{2w^{T}Vw}-\log\frac{\eta }{1-\eta }$$

Note that *P*(*D* = 1| *y*) is a monotonically increasing function of *y* if *A* < 0, i.e., *w^T^*(*μ*_1_ – *μ*_0_) > 0.
